# Sequential Neural Activity in Primary Motor Cortex during Sleep

**DOI:** 10.1523/JNEUROSCI.1408-18.2019

**Published:** 2019-05-08

**Authors:** Wei Xu, Felipe de Carvalho, Andrew Jackson

**Affiliations:** Institute of Neuroscience, Newcastle University, Newcastle NE2 4HH, United Kingdom

**Keywords:** motor cortex, nonhuman primate, sequential activity, sleep, theta bursts

## Abstract

Sequential firing of neurons during sleep is thought to play a role in the consolidation of learning. However, direct evidence for such sequence replay is limited to only a few brain areas and sleep states mainly in rodents. Using a custom-designed wearable neural data logger and chronically implanted electrodes, we made long-term recordings of neural activity in the primary motor cortex of two female nonhuman primates during free behavior and natural sleep. We used the local field potential (LFP) spectrogram to characterize sleep cycles, and examined firing rates, correlations, and sequential firing of neurons at different frequency bands through the cycle. Slow-wave sleep (SWS) was characterized by low neural firing rates and high synchrony, reflecting slow oscillations between cortical down and up states. However, the order in which neurons entered up states was similar to the sequence of neural activity observed at low frequencies during waking behavior. In addition, we found evidence of brief bursts of theta oscillation, associated with non-SWS states, during which neurons fired in strikingly regular sequential order phase-locked to the LFP. Theta sequences were preserved between waking and sleep, but appeared not to resemble the order of neural activity observed at lower frequencies. The sequential firing of neurons during slow oscillations and theta bursts may contribute to the consolidation of procedural memories during sleep.

**SIGNIFICANCE STATEMENT** Replay of sequential neural activity during sleep is believed to support consolidation of daytime learning. Despite a wealth of studies investigating sequential replay in association with episodic and spatial memory, it is unknown whether similar sequences occur in motor areas during sleep. Within long-term neural recordings from monkey motor cortex, we found two distinct patterns of sequential activity during different phases of the natural sleep cycle. Slow-wave sleep was associated with delta-band sequences that resembled low-frequency activity during movement, while occasional brief bursts of theta oscillation were associated with a different order of sequential firing. Our results are the first report of sequential sleep replay in the motor cortex, which may play an important role in consolidation of procedural learning.

## Introduction

Brain activity during sleep is implicated in stabilizing, consolidating, and reorganizing daytime learning, but the neural mechanisms remain unclear. One influential theory states that the reactivation of specific sequences of neural firing observed during the day (sleep “replay”) drives synaptic changes through spike-timing-dependent plasticity, which depends critically on the temporal order of presynaptic and postsynaptic activity (Kruskal et al., 2013). Sequential replay was first reported in the hippocampus of sleeping rats (Wilson and McNaughton, 1994), and has since been observed the striatum (Pennartz et al., 2004) and prefrontal cortex (Euston et al., 2007; Peyrache et al., 2009), spawning a wealth of literature concerning the consolidation of spatial and rule learning. However, while sleep is also implicated in consolidation and off-line gains following motor learning (Walker et al., 2002; Nitsche et al., 2010; Tucker et al., 2017), there has been considerably less investigation of single-unit activity in motor areas during sleep. Synchronous reactivation in rodent slow-wave sleep has been linked to consolidation of forelimb reaching behaviors (Ramanathan et al., 2015), but there have been no reports of sequential firing similar to that observed in the episodic memory system. We have previously shown in monkeys that delta oscillations in local field potentials (LFPs) during anesthesia and slow-wave sleep share a common structure with 2–3 Hz cortical cycles during awake movements (Hall et al., 2014a). Since neural firing rates exhibit consistent cyclical structure at these frequencies across different awake behaviors (Churchland et al., 2012; Russo et al., 2018), we speculated that the same sequences might also occur at delta frequencies during cortical cycles in slow-wave sleep. In addition, we sought evidence for sequential activity at other frequencies during other phases of the natural sleep cycle.

To examine patterns of neural correlation across the sleep cycle, we used a wearable data logger for recording long-term multichannel spiking activity in the motor cortex of monkeys during free behavior and natural sleep. During slow-wave sleep, the dominant pattern was broad synchrony between neurons reflecting the slow oscillation between cortical down and up states. However, neurons entered the up state at slightly different times, leading to sequential activation at low frequencies. This order resembled that seen during awake behavior, suggesting that the same intrinsic dynamics govern both states. Outside of slow-wave sleep, and occasionally in awake states, we observed brief epochs of theta oscillation during which neurons fired rhythmically in consistent sequences. Interestingly, the order of neural firing during theta bursts was unrelated to that seen at lower frequencies, and we suggest that this represents a different network state that may be well-suited to drive neuroplasticity of motor cortical circuits.

## Materials and Methods

### 

#### Wearable neural data logger

**Figure 1.**
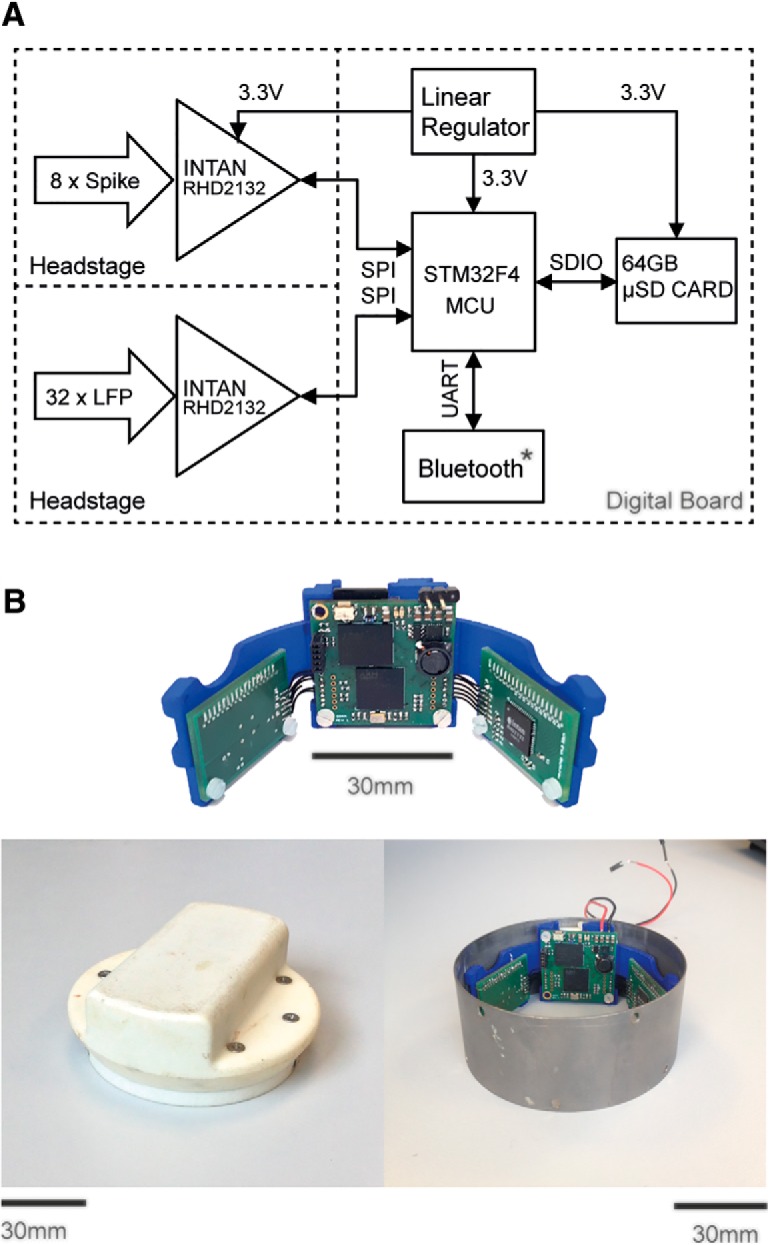
Wearable neural data logger. ***A***, System schematic showing main components including two RHD2132 amplifiers (Intan Technologies) amplifiers connecting via a serial peripheral interface (SPI) to a STM43F4 microcontroller unit (MCU; STMicroelectronics). ***B***, Top, Neural data logger printed circuit boards fixed to the support cradle. Bottom right, The device fitted inside a titanium headpiece similar to the one used in the monkeys. Bottom left, The lid holds the battery and protects the electronics and connectors. The total weight of the electronics plus battery is 152 g.

We developed a custom wearable neural data logger (Fig. 1). This enabled us to record long-term neural activity during wake and sleep in nonhuman primates without the constraints imposed by physical tethers or the limited transmission ranges and battery lifetimes associated with wireless telemetry. The device incorporates two multichannel bioamplifiers (gain, ×192; Intan Technologies, RHD2132) configured to record eight channels of wideband neural signals (0.1 Hz–7.5 kHz bandwidth, 20 kHz sampling rate) and 32 channels of LFP signals (0.1–300 Hz bandwidth, 1 kHz sampling rate). The 16-bit digitized samples were sent via a serial peripheral interface to a low-power microcontroller (STMicroelectronics, STM32F407), which packaged and relayed the data in time-stamped 16 KB blocks to a 64 GB microSD card.

The neural logger was implemented on three printed circuit boards (one 30 × 30 mm digital board and two 30 × 20 mm headstages), which were mounted inside a titanium head casing that also contained a 3.7 V, 5200 mAh rechargeable battery. This system was capable of recording for >24 h, thereby providing continuous monitoring of neural data with only a daily replacement of the microSD card and battery.

#### Surgical procedures

Experiments were approved by local ethics committee and performed under appropriate United Kingdom Home Office licenses in accordance with the Animals (Scientific Procedures) Act of 1986. Two purpose-bred female rhesus macaques (O: 9 years old, 6.8 kg; U: 6 years old, 7.2 kg) were used for this study. Surgeries were performed in sterile conditions under sevoflurane anesthesia with appropriate postoperative analgesics and antibiotics. The animals were implanted with custom arrays comprising 12 moveable 50-μm-diameter tungsten microwires (impedance, ∼200 kΩ at 1 kHz) and four 16-channel linear microelectrode arrays (LMAs; Microprobe). Our moveable microwire array, described in detail previously (Jackson and Fetz, 2007), allows electrodes to be individually positioned. We find that these arrays yield stable recordings of the same single units over multiple days to several months. The LMAs were incorporated to provide depth profiles of LFPs, and comprised 16 evenly spaced contacts (separation: 250 μm for short LMAs, 500 μm for long LMAs). Monkey O received two such combined arrays, implanted bilaterally in the primary motor cortex (M1). Monkey U received a single array implanted in the right M1. Implantation was guided by a prior structural MRI scan and intraoperative identification of the central sulcus.

Additionally, Monkey U was implanted with electromyogram (EMG) electrodes in six left-arm muscles (extensor carpi radialis, extensor carpi ulnaris, flexor carpi radialis, flexor carpi ulnaris, biceps, triceps), comprising pairs of insulated stainless steel wire (Cooner, AS632) sutured to the muscle fascia and routed subcutaneously to the head-mounted titanium casing that contained connectors, electronics, and a battery.

#### Home-cage recordings

Overnight recordings were taken following conventional recording sessions in the laboratory, 4 d per week, over a period of 5 months. Here we report only data from recordings that captured a full night of sleep and were not corrupted by excess noise (large artifacts or line noise associated with broken connections). Recordings usually began in the late afternoon and lasted on average 20.9 ± 3.8 h (46 nights for Monkey O) and 20.4 ± 2.3 h (99 nights for Monkey U). Variations in the recording period are accounted by different lengths of laboratory sessions and different start times for those sessions.

#### Spike sorting and spike discrimination

**Figure 2.**
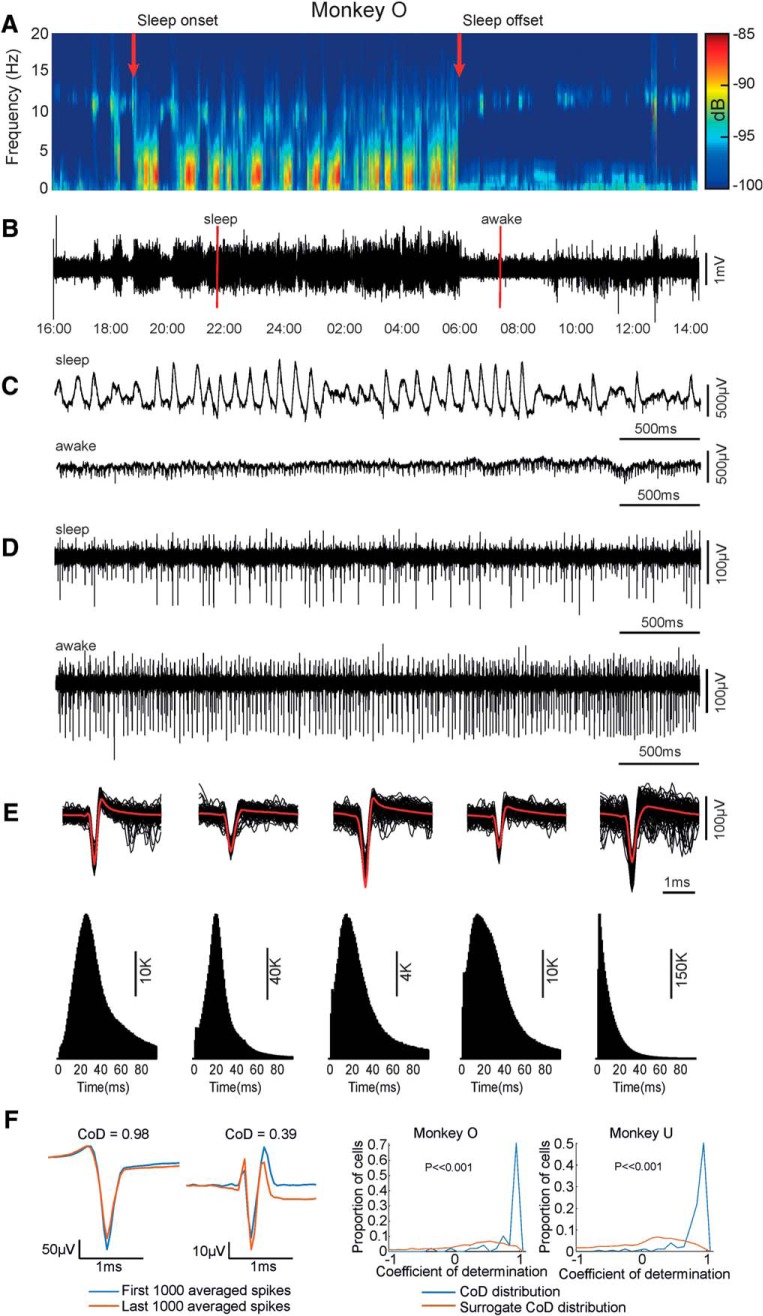
Example motor cortex spike and LFP signals for a typical overnight recording. ***A***, Time–frequency spectrogram of LFP from a single cortical electrode. ***B***, LFP waveform for the entire recording period. ***C***, 4 s windows showing LFP during sleep (top trace) and awake (lower trace) periods. The two time points corresponding to these traces are indicated with red lines in ***B***. ***D***, The same LFP sections as in ***B***, after bandpass filtering (1–8 kHz) to show action potentials. ***E***, ISI histograms (1 ms bin width) and action potential waveforms for four single-unit and one multiunit spike trains discriminated in a single dataset. ***F***, Left, Average waveform of the first and last 1000 spikes for two example sessions. Waveform stability is indicated by high CoDs. Right, Histogram of CoD for all cells (blue trace). Also shown is the surrogate distribution generated by comparing waveform similarity of different neurons in the recording (red trace).

All analyses were performed in Matlab (Mathworks). Data were initially decoded and saved to a computer using custom scripts. Spike discrimination was performed offline using Wave_clus (Quiroga et al., 2004), configured with a 1–8 kHz bandpass filter and an amplitude threshold of four SDs above signal mean for spike detection. From the 12 microwire electrodes, we were able routinely to discriminate several single-unit and multiunit spikes per recording session [example spike waveforms and interspike interval (ISI) histograms are shown in Fig. 2*E*]. Analysis is based only on single units exhibiting a clear peak in the ISI histogram. We obtained 0–7 such units per session. For Monkey O, we recorded 70 M1 single units. For Monkey U, we recorded 184 single units.

#### Experimental design and statistical analysis

##### Assessing stability of spike waveforms.

To verify that the Neurochip was able to record the same neurons throughout these long sessions, we assessed the similarity between the average waveforms of the first 1000 spikes and last 1000 spikes within each session using a coefficient of determination (CoD; Eq. 1):

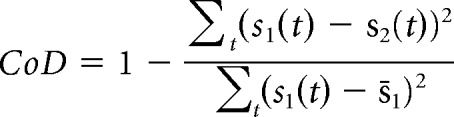
 where *s*_1_(*t*) and *s*_2_(*t*) are the mean waveforms at the start and end of the session, and s̄_1_ is the mean value of *s*_1_(*t*). Note that a CoD close to 1 indicates a similar waveform, suggesting that same neuron has been maintained throughout. To compare our experimental CoD values against what would be expected if the neurons were not the same at the beginning and end of recordings, we bootstrapped the distribution of CoD values obtained by comparing spike waveforms recorded at the beginning of sessions with spike waveforms for a different neuron at the end of recordings. We performed 1000 iterations of this shuffling procedure to test the overall significance of our mean CoD value.

##### Defining sleep periods.

Data were down-sampled to 250 Hz before performing a fast Fourier transform (FFT) using a 512-point (2.048 s) window. We defined sleep periods based on the onset and offset of high-amplitude LFP activity averaged over 5 min windows (assessed by eye from power spectrograms; Fig. 2*A*), and validated these judgements in Monkey O with simultaneously acquired video recordings. The times of falling asleep and waking up judged from electrophysiological and video recordings were not statistically different (paired *t* test, *p* = 0.57 and 0.91 for sleep onset and offset respectively, *n* = 22).

##### Defining sleep-cycle phase.

**Figure 3.**
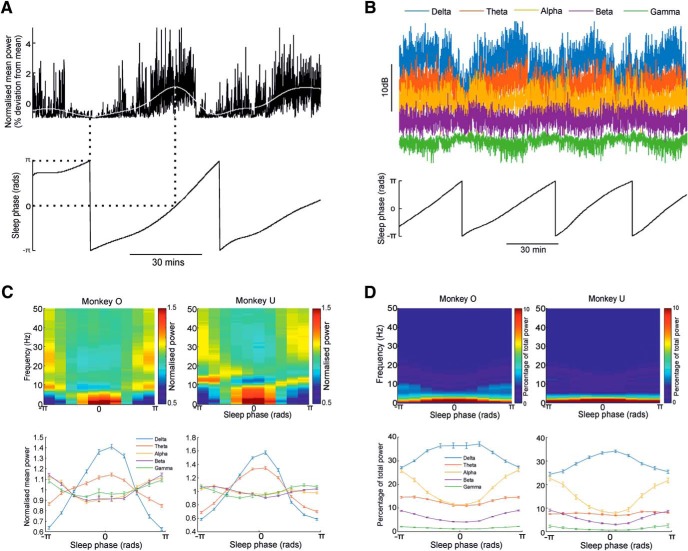
LFP power modulation through the sleep cycle. ***A***, LFP power <1 Hz (top, black line) was smoothed (top, white line) and used to derive the instantaneous phase of sleep via a Hilbert transform (bottom). ***B***, LFP power in different frequency bands and associated sleep phase. ***C***, LFP power averaged through the sleep cycle (normalized as a proportion of the mean power across the sleep cycle at that frequency). ***D***, LFP power averaged through the sleep cycle (normalized as a proportion of the mean power across all frequencies for that sleep phase).

Conventionally, human sleep periods have been separated into different phases by visual examination of the EEG (Berry et al., 2015). Different sleep phases are associated with relative increases or decreases in power at different frequency bands of the EEG and/or LFP (Iber et al., 2007; Destexhe et al., 1999), but there is no consensus on the number of identifiable discrete sleep states in animals (Kleinlogel, 1990; Gottesmann, 1992). To provide a simple and consistent characterization of the sleep cycle, we used the periodic fluctuations in the power of low-frequency (<1 Hz) LFPs (Achermann and Borbély, 1997; Steriade et al., 1993a,b; Destexhe et al., 1999) to derive a continuous measure of sleep phase (Fig. 3*A*). We low-pass filtered and averaged the low-frequency power in all channels, and applied a Hilbert transform to extract an instantaneous phase that varied from −π to π (where zero-phase corresponds to maximal low-frequency power, i.e., slow-wave sleep). Sleep phase was divided into 10 equal bins for subsequent cycle-aligned analyses of activity patterns.

##### Analysis of firing rates through the sleep cycle.

The modulation of average firing rates binned by phase through the sleep cycle was assessed using a circular–linear correlation coefficient (Eq. 2):

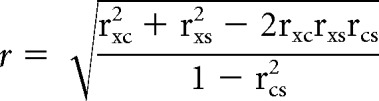
 where r_xc_, r_xs_, and r_cs_ are the Pearson's correlation coefficients between, respectively, firing rate versus cosine of sleep phase, firing rate versus sine of sleep phase, and cosine of sleep phase versus sine of sleep phase (which equals zero for equally spaced phase bins). Significance testing was performed using the circ_stats package in Matlab.

##### Time–domain correlation analyses.

The recording period was divided into consecutive 5 min windows. Within each window, we calculated spike-triggered averages (STAs) of LFPs and cross-correlation histograms (CCHs) between simultaneously recorded spike trains (using 10-ms-wide bins). CCHs were normalized and expressed as the proportion of excess spike pairs relative to that expected for uniform firing with the same mean rate as the actual neurons within each 5 min window. STAs and CCHs were then averaged over all 5 min windows during wake/sleep periods, or divided according to the phase of sleep that each window occurred in each window. The strength of spike–LFP correlation was measured as the peak-to-peak amplitude of the STA, while the strength of spike–spike correlation was measured as the zero-lag amplitude of the CCH.

##### Spike–field coherence.

Spike times were converted into quasicontinuous firing rate signals by binning spikes into 4 ms bins, corresponding to the sampling interval of LFPs at 250 Hz. Standard coherence analysis between firing rates and LFPs, *Coh_spike–LFP_*, was calculated as the magnitude-squared of the normalized complex cross-spectrum, *X_spike–LFP_* (Eq. 3):

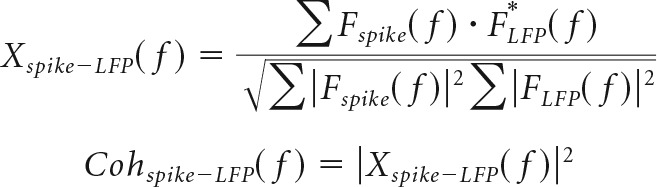
 where *F_spike_*(*f*) and *F_LFP_*(*f*) are the 512-point FFT coefficients of the spike rate and LFP at frequency *f*, * denotes complex conjugation, and the summation is performed over all windows within a particular interval of the sleep cycle.

##### Spike–spike coherence.

As with spike–LFP analyses, we also calculated the normalized cross-spectrum between the firing rates of pairs of spikes (Eq. 4):

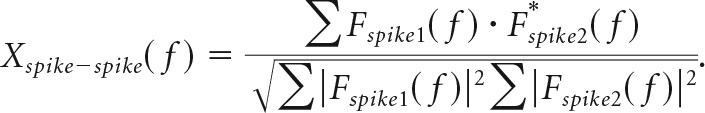


The phase of the cross-spectrum reflects the phase difference between neural activity. Thus, a real value implies in-phase/antiphase firing while a purely imaginary value implies a 90° phase shift between neurons. It is convenient to decompose the total coherence between spike trains into real and imaginary components (Eq. 5):

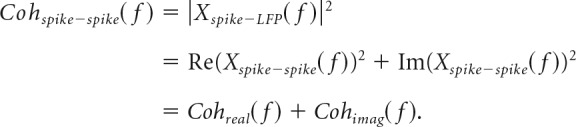


Note that statistically significant, nonzero imaginary coherence, *Coh_imag_*(*f*), implies that there is a consistent phase order to neural firing at frequency *f*, although that phase difference not need to be exactly 90°. Nonparametric significance testing of imaginary coherence was carried by bootstrapping the expected distribution of imaginary coherence between neurons in the absence of a consistent phase order between them. To achieve this, we used the same Fourier amplitudes as the real data but randomly shuffled the phases of Fourier coefficients across neurons on a window-by-window basis. We repeated this processes 1000 times and calculated the 95th percentile value of the resultant distribution.

##### Sequence similarity.

The sign of the (unsquared) imaginary component of coherence, Im[*X*_spike–spike_(*f*)], reflects the order of sequential firing between neurons. This allows comparison of the sequence structure between different behavioral states (e.g., wake vs sleep). A conserved sequence (e.g., 1–>2–>3) would be one in which the imaginary coherence between all neuron pairs ([1,2], [2,1], [1,3], [3,1], [2,3], [3,2]) would have the same sign (+, −, +, −, +, −, respectively) in both behavioral states. Therefore, we used correlation analysis over all neuron pairs between the different states as a measure of sequence preservation. Note that the imaginary coherence between different pairs of neurons within the same recording cannot be treated as independent observations (trivially, for sessions with two neurons *Coh_imag_*[1,2] = −*Coh_imag_*[2,1], while the number of pairwise values grows combinatorially as the number of independent neurons increases). Therefore, we used a nonparametric approach to significance testing, based on bootstrapping the expected distribution of correlation values after shuffling the labeling of neurons (not neuron pairs) within the dataset corresponding to one of the behavioral states. Shuffling was performed within each recording session and across sessions that contained the same number of neuron pairs. In this way, we were able to bootstrap the expected range of correlation values from 1000 surrogate datasets of pairwise imaginary coherence values with the same statistical distribution as the original data but no systematic relationship between behavioral conditions.

## Results

### Continuous long-term recording during waking and sleep

Our wearable neural data logger (Fig. 1) provided stable, long-term recordings from multiple motor cortical electrodes in unrestrained monkeys. Figure 2*A,B* shows example LFP data from a single electrode collected over a 22 h recording period, including awake home-cage behavior and natural sleep. At expanded time resolution (Fig. 2*C*), spiking activity is evident on top of the LFP, and this is revealed more clearly after high-pass filtering (>1 kHz; Fig. 2*D*). Example waveforms and ISI histograms for all single-unit and multiunit activity captured in this recording session are shown in Figure 2*E*.

To ensure we were recording the same individual neurons across such long sessions, we assessed the similarity between average spike waveforms at the beginning and end of recordings (Fig. 2*F*, *left*) using a CoD. Values close to 1 indicate that we were able to maintain highly stability recordings (Fig. 2*F*, right). To ensure that these high CoD values were not simply the result of general features shared by all neurons, we calculated the distribution of CoDs between waveforms of different neurons in our dataset. CoDs for the same neuron at the beginning/end of recordings were significantly greater than CoDs between different neurons (*p* < 0.001 for both animals).

### LFP oscillations across the sleep cycle

Evident in the LFP time–frequency spectrogram (Fig. 2*A*) is a cyclical variation in LFP power during the night, between periods of slow-wave activity and periods of higher-frequency oscillation. To characterize these sleep cycles, we used the time-varying low-frequency (<1 Hz) power (Fig. 3*A*) to define an instantaneous phase that varied from −π to π (where zero-phase corresponds to maximal slow-wave activity). The mean (±SE) duration of sleep cycles was 60 ± 3 min (Monkey O, 46 sessions) and 55 ± 1 min (Monkey U, 99 sessions), which is consistent with previously reported sleep cycles of 40–60 min for macaques (Campbell and Tobler, 1984; Jackson et al., 2007). The mean power of <1 Hz was significantly lower in the second half of the sleep duration compared to the first (paired *t* test, Monkey O: *p* = 0.003; Monkey U: *p* = 2.20e-9), in keeping with a homeostatic decrease in sleep pressure (Vyazovskiy et al., 2007).

Figure 3*B* shows example modulation of power in other LFP bands corresponding to delta (1–4 Hz), theta (4–7 Hz), alpha (7–15 Hz), beta (15–31 Hz), and gamma (>31 Hz) bands. Using the instantaneous measure of sleep phase derived from <1 Hz power, we averaged the modulation of power at other frequencies over the sleep cycle. We normalized power either as a proportion of the average power at that frequency across the sleep cycle (Fig. 3*C*), or as a proportion of total power across all frequencies for that sleep phase (Fig. 3*D*). These sleep cycle-averaged power spectrograms revealed clearly the reciprocity between LFP activity at low (<4 Hz) frequencies associated with phases of maximal slow-wave activity, and high (>7 Hz) frequencies during lighter sleep phases. For the theta (4–7 Hz) band, absolute power was highest during slow-wave phases (Fig. 3*C*), but this represented a smaller proportion of total power than during lighter sleep (Fig. 3*D*).

### Single-unit firing rates and EMG activity across the sleep cycle

**Figure 4.**
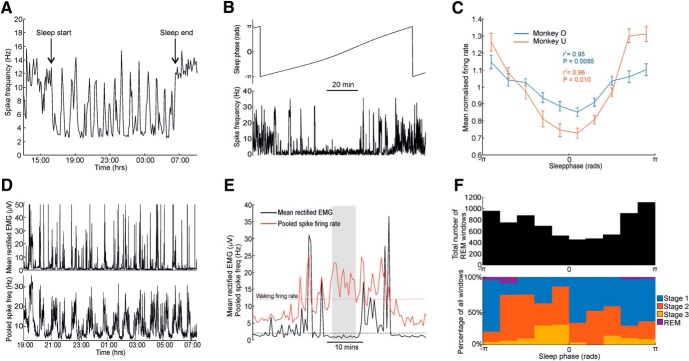
Motor cortex firing rates during sleep. ***A***, Mean firing rate for an example neuron across the duration of an entire recording (divided into 5 min windows) showing periodic firing-rate fluctuations during sleep. ***B***, Firing rate (calculated over 2 s windows) for the same cell across a single sleep cycle. ***C***, Mean normalized (relative to mean rate) firing for all cells and all sessions. A significant modulation with sleep cycle is demonstrated using circular-to-linear correlation. ***D***, Firing of an example neuron together with simultaneously recorded EMG during the night. ***E***, Example of period of putative REM sleep (indicated by shading) where spike firing is at frequencies higher than mean waking rates but not associated with muscle activity. ***F***, Top, Histogram of the instantaneous sleep phases corresponding to all putative REM windows. Bottom, Results of manually sleep-scored sample windows taken from different sleep phases.

The firing rates of single neurons also varied during the sleep cycle. For the example neuron shown in Figure 4*A*,*B*, firing decreased during slow-wave sleep (corresponding to sleep phases around 0). Average normalized firing rates across the sleep cycle (using our instantaneous sleep-phase measure) revealed this to be the dominant pattern for most neurons (Fig. 4*C*). High firing rates observed outside slow-wave sleep are indicative of rapid eye movement (REM) periods, which are characterized by brain activity resembling the waking state but profound paralysis of muscles (Brooks and Peever, 2012; Steriade and McCarley, 1990). For Monkey U, we were able to obtain simultaneous EMG recordings from six muscles in the left forearm during sleep via implanted EMG electrodes. We were also able to obtain from the same monkey simultaneous single-unit spiking in the M1 (Fig. 4*D*). As reported previously (Jackson et al., 2007), periods of high cortical firing rates were often associated with profoundly suppressed EMG (Fig. 4*E*). We identified putative REM sleep as any 30 s periods in which firing rates exceeded average waking levels while average rectified EMG was <2 μV (Berry et al., 2015). Such windows tended to fall more often around instantaneous sleep phase values of π and −π (Fig. 4*F*, top). Finally, we validated our sleep-phase measure by having 300 randomly selected 10 s sections of LFP and EMG data from Monkey U scored manually by an experienced EEG sleep technician blinded to our results (Fig. 4*F*, bottom). Again, REM sleep was associated with sleep phases of π and −π, while sleep phases of zero tended to be scored as stage 2 and 3 of slow-wave sleep. This further supported our use of the instantaneous phase of slow-wave power as a simple yet robust method of characterizing the sleep cycle in nonhuman primates.

### Correlations between spikes and LFPs during wake and sleep

**Figure 5.**
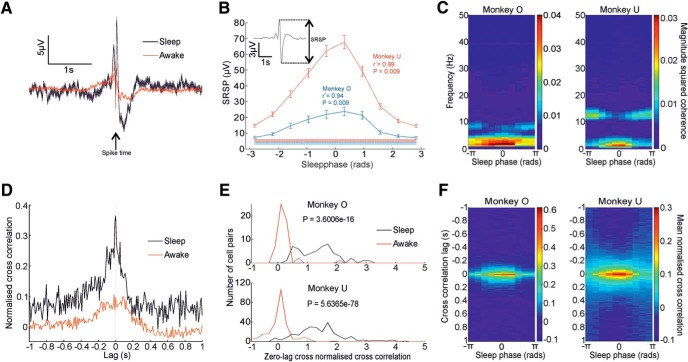
Spike–LFP and spike–spike correlations during wake and sleep. ***A***, Spike-triggered average of an LFP showing a similar profile but increased magnitude during sleep versus awake periods. Shaded regions represent SE. ***B***, Mean SRSP magnitude against sleep phase for all cells and all sessions for both monkeys. Horizontal lines represent mean ± SEM of magnitudes during waking. *r* and *P* values derived from circular-to-linear correlation analysis. ***C***, Mean magnitude squared coherence between spikes and LFPs for frequencies ≤50 Hz as a function of sleep phase. ***D***, Normalized CCH for an example cell pair during all wake (red line) and sleep (black line) periods of the recording. ***E***, Strength of zero-lag correlations between all cell pairs during wake and sleep. ***F***, Mean normalized CCH as a function of sleep phase.

To investigate how the dynamics of the neural population varied across the sleep cycle, we first examined the relationship of single-neuron spiking to the LFP. Previously, we have shown that spike-triggered average LFPs reveal a low-frequency component [termed the spike-related slow potential (SRSP)] with a similar shape in both waking and light-sleep states (Hall et al., 2014a,b). Note that the biphasic shape may be due to causal filtering by the amplifiers of a monophasic trough (Okun, 2017). SRSPs were also evident in our overnight recordings (Fig. 5*A*), with a magnitude that varied systematically through the sleep cycle (Fig. 5*B*) and was largest during slow-wave sleep. Spike-field coherence (Fig. 5*C*) confirmed strong phase-locking at slow oscillation frequencies during slow-wave sleep, as well as weaker coherence at alpha frequencies (7–15 Hz) during nonslow-wave phases.

Next we examined correlations between pairs of neurons using normalized CCHs (Fig. 5*D*; see Materials and Methods). The majority of zero-lag cross-correlations were positive during sleep (100% for Monkey O; 97% for Monkey U; Fig. 5*E*) and significantly greater than during waking (*p* < 0.05, paired *t* test for both monkeys). Correlated neural firing was also modulated across the sleep cycle, with the highest synchrony occurring during slow-wave sleep (Fig. 5*F*).

In summary, motor cortical neurons are broadly synchronized during slow-wave sleep, and strongly phase-locked to low-frequency LFP. The shape of the SRSP suggests neurons are maximally active during the negative-going LFP. This likely reflects the known relationship between cortical up/down states (characterized by high/low firing rates, respectively) and the slow oscillation observed in the LFP (Steriade et al., 1993b, 2001). Outside slow-wave sleep, neural activity is less synchronized, but weakly phase-locked to LFP activity in the alpha frequency band (7–15 Hz), which may correspond to sleep spindles.

### Sequential neural firing during slow-wave sleep

**Figure 6.**
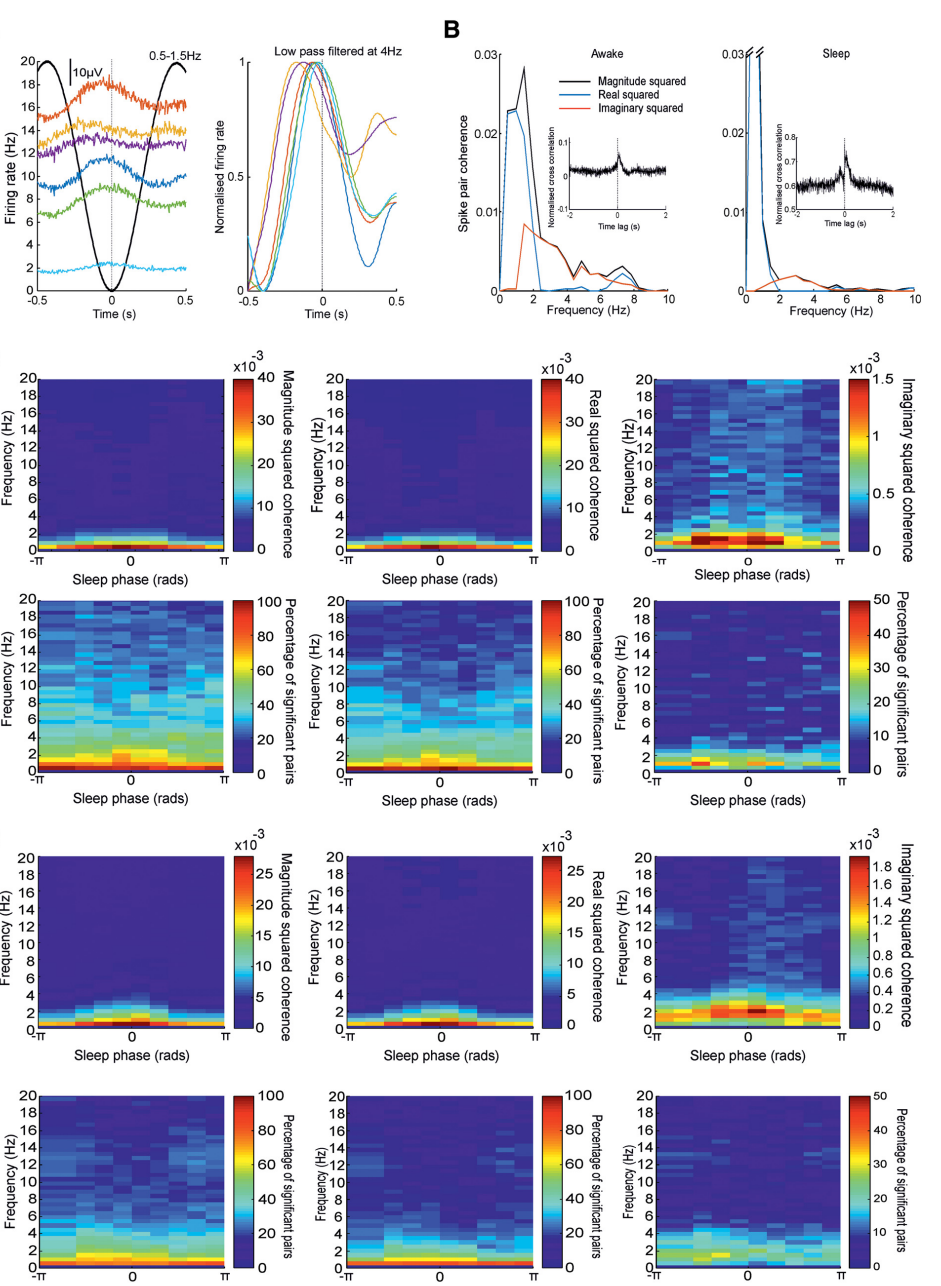
Real and imaginary coherence between cell pairs. ***A***, Average raw (left) and filtered/normalized (right) spike firing frequency aligned to the trough of low-frequency LFP for six neurons recorded in a single session. ***B***, Magnitude squared, real squared, and imaginary squared coherence for an example pair of neurons during sleep (left) and waking (right). ***C***, ***D***, Top row, Total, real, and imaginary squared coherence for all cell pairs averaged across the sleep cycle for both monkeys. Bottom row, Proportion of cell pairs exhibiting significant (*p* < 0.05) coherence at each frequency across the sleep cycle.

Although the dominant pattern of correlation between neurons during sleep was broadly synchronous, we examined whether there was evidence for sequential activity at any frequency. Since spike-triggered averages suggested that neural firing was modulated with the slow LFP oscillation, we first examined the firing rates of multiple neurons aligned to the trough of the low-frequency LFP. The example in Figure 6*A* clearly shows an increase in firing rates (the up state) occurring during the falling phase of the slow LFP oscillation. Note, however, that this firing-rate increase occurs at a slightly different latency for each neuron, leading to a sequential transition into the up state.

A convenient way to quantify such sequential firing in the frequency domain uses the imaginary coherence between pairs of spike trains. Conventional coherence is expressed as the magnitude squared of the (complex) normalized cross-spectrum, but this can be decomposed into the sum-squared of real (in-phase/antiphase) component and imaginary (quadrature-phase) components. Nonzero imaginary coherence implies that for a given frequency, the activity of one neuron consistently lags or leads the other (although not necessarily by exactly 90°). Figure 6*B* shows the decomposition of coherence into real and imaginary squared components for an example spike pair. At very low frequencies, coherence was dominated by a large, real component reflecting correlated in-phase modulation. However, a peak in imaginary coherence around 2–3 Hz revealed consistent sequential activity in the delta band. Note that sequential firing at this frequency was observed during both waking and sleeping states.

We next examined how real and imaginary coherence between neurons was modulated through the sleep cycle (Fig. 6*C*). Real squared coherence was dominant at low frequencies and was highest during slow-wave sleep. Additionally, during slow-wave sleep, a statistically significant (albeit weak) imaginary component at delta frequencies was observed, indicating sequential activation through the slow oscillation.

### Sequential firing during theta bursts

**Figure 7.**
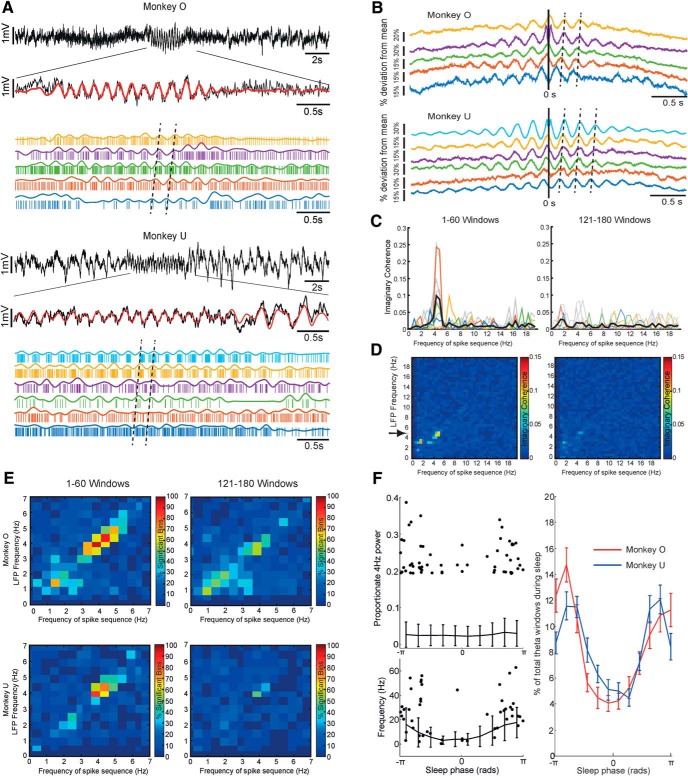
Sequential firing during theta bursts. ***A***, Examples of LFP theta bursts with corresponding neural spiking shown in the expanded traces below. Note that different neurons are plotted in order of firing during the theta cycle. ***B***, Example CCHs during periods of high LFP theta. Cross-correlations for all cells relative to a single reference cell are shown. ***C***, Left, Imaginary coherence between spike trains during the top 60 windows of highest LFP theta. Colored lines correspond to the CCHs shown above (Monkey O), while gray lines show all other cell pairs in this recording. The black trace shows the average over cell pairs. Right, The corresponding analysis applied to windows ranked 121–180th in LFP theta. ***D***, Mean imaginary coherence for the same session computed over windows sorted according to a range of LFP frequencies (0–20 Hz). ***E***, Proportion of all datasets exhibiting significant imaginary coherence at each firing-rate frequency, when calculated over windows sorted according to all LFP frequencies. Significant sequential firing at theta frequencies is a consistent finding, but only for windows exhibiting high LFP theta. ***F***, Left, Solid lines indicate mean theta power and mean spike firing rate as a function of sleep and high theta power. Error bars represent SD. Dots indicate theta power and firing rate associated with the top 60 windows of highest LFP theta for an example session in Monkey U. Right, Sleep phase associated with the top 60 windows of highest LFP theta over all sessions.

The previous analysis suggests that when averaged across the entire day or night, sequential firing of neurons (indicated by imaginary squared coherence) is weak and limited to the delta band. However, visual inspection of the raw data revealed occasional distinctive bursts of highly rhythmical theta-band oscillations at around 4–5 Hz (Fig. 7*A*). During these theta bursts, multiple neurons were phase-locked to the oscillation but fired in sequence with different preferred phases.

To quantify this phenomenon, we focused our analysis on only 60 time windows (length, 2.048 s) from each recording that had the highest relative (proportion of total) LFP power at theta frequencies. CCHs compiled between spike trains for these theta burst windows showed clear oscillatory correlations, with peaks at nonzero time lags revealing sequential activation (Fig. 7*B*). As before, we used the imaginary coherence between spike trains to assess the consistency of the sequence structure within theta bursts. Clear peaks around 4–5 Hz were evident when we restricted our analysis to only the 60 time windows characterized by highest relative theta LFP power (Fig. 7*C*, left). Note that such sequential theta firing was a relatively rare occurrence throughout a single recording. We chose 60 windows because including more windows tended to reduce the strength of imaginary coherence at theta frequencies. When we analyzed a different subset of 60 windows ranked 121–180th in relative theta power, no imaginary coherence was observed (Fig. 7*C*, right). Analysis of windows ranked 61–120th in relative theta power yielded inconsistent results with only small peaks in imaginary coherence in some sessions.

Next, we examined whether this sequential firing phenomenon was restricted to the theta band, or if it could be observed during epochs of LFP oscillation at other frequencies. Therefore, we repeated our analysis of imaginary coherence using 60 windows selected to have the highest relative LFP power at a range of frequencies ≤20 Hz. The results for one session are shown in the two-dimensional color plot on the left of Figure 7*D*. The vertical axis shows the LFP frequency selected, while the horizontal axis shows the frequency of spike–spike imaginary coherence. The dominant feature is a peak in theta coherence, occurring only when windows are selected for high theta LFP power. As before, this peak disappeared when windows ranked 121–181th in relative LFP power were analyzed (Fig. 7*D*, right). We summarized the entire dataset by plotting the proportion of statistically significant imaginary coherence values in each frequency bin across all sessions for both animals (Fig. 7*E*; see Materials and Methods). Sequential firing during theta bursts was a consistent finding in most sessions, occurring most commonly during windows selected for high LFP power at 4 Hz. Although theta power during these selected windows was ≥4 times greater than average, firing rates were only slightly higher than those of other windows at the same sleep phase. Theta bursts predominantly occurred during nonslow-wave sleep around sleep phases of ±π (Fig. 7*F*), consistent with REM and/or light-sleep states, which in humans are characterized by theta oscillation. Note, however, that a proportion of theta bursts in both animals also occurred during periods of awake recording (14% for Monkey O; 29% for Monkey U).

### Sequential orders are preserved across sleeping and waking periods

**Figure 8.**
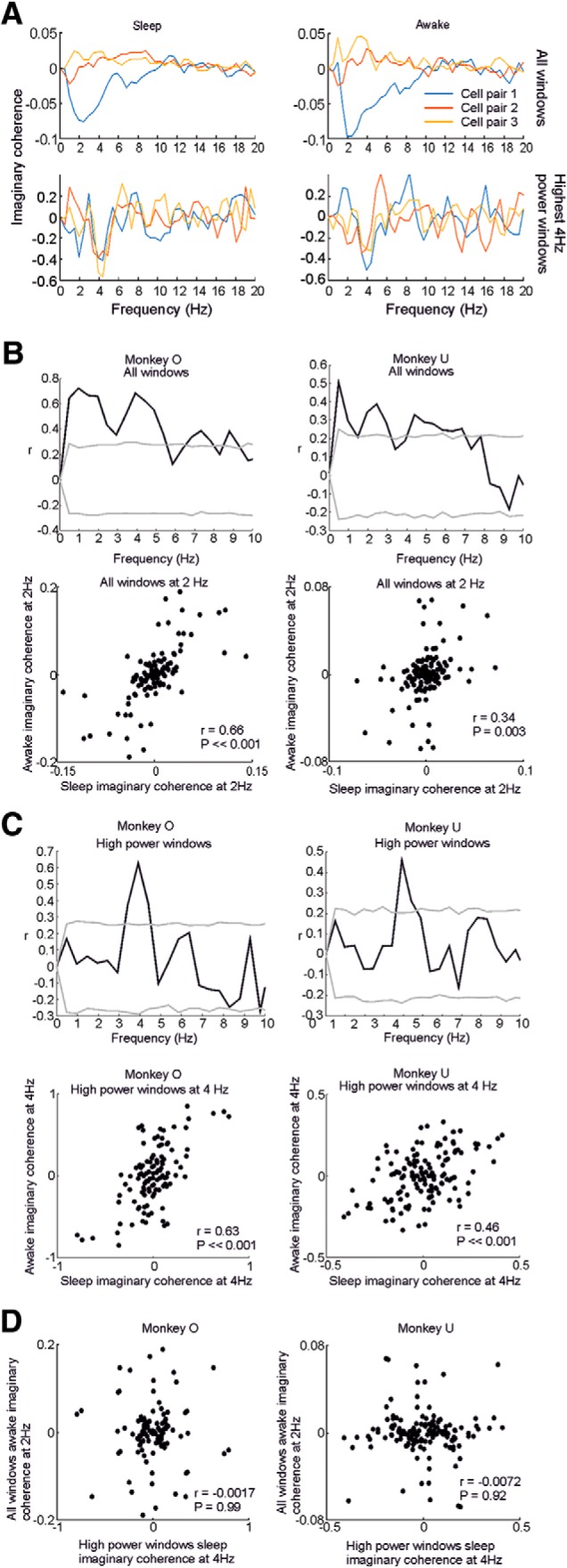
Consistent sequential firing between sleep and waking. ***A***, Signed imaginary coherence spectra for three example neuron pairs during sleep (left) and waking (right) periods, calculated for all windows (top) and during theta bursts (bottom). ***B***, Top row, Correlation coefficient between pairwise imaginary coherence values in wake and sleep (all windows), across all neuron pairs for both monkeys. Gray lines indicate 95% significance thresholds obtained by shuffling across neurons. Bottom row, Scatter plot of sleep versus awake imaginary coherence at an example frequency of 2 Hz. ***C***, Top row, Correlation coefficient between pairwise imaginary coherence values in wake and sleep (during theta bursts), across all neuron pairs for both monkeys. Gray lines indicate 95% significance thresholds obtained by shuffling across neurons. Bottom row, Scatter plot of sleep versus awake imaginary coherence at an example frequency of 4 Hz. ***D***, Scatter plot of imaginary coherence values at 4 Hz (during theta bursts) against imaginary coherence at 2 Hz (during all waking periods).

The unsquared imaginary coherence between pairs of spike trains provides an indication of not only the strength (magnitude), but also the order (sign) of sequential firing. This enabled us to examine whether the sequence of firing was preserved at given frequencies between waking and sleep. We first assessed whether any sequential order was preserved between the entire durations of wake and sleep periods. Figure 8*A* (top row) shows example signed imaginary coherence spectra for three cell pairs during waking and sleep. If there were no relationship between sequential firing in wake and sleep, we would expect the sign of imaginary coherence in each state to be unrelated. However, the strong resemblance between delta frequency components in each condition (negative, positive, positive, respectively, for pairs 1, 2, and 3) suggests that if pairs of cells that fire in consistent order during sleep also tend to fire in the same order during waking.

We also calculated signed imaginary coherence spectra for only those 60 windows (in either waking or sleep) that had the highest relative LFP power at theta frequency (Fig. 8*A*, bottom row). In this case a (negative) trough was observed for all three cell pairs in each condition. This again suggests a conserved order of firing between waking and sleep, but one that is different during theta bursts to that seen at delta frequencies. Note that this difference can also be seen by comparing the examples in Figures 6*A* and 7*A* (lower plot), which come from the same session and have the same color-coding of neurons. The order of firing during the up state (Fig. 6*A*) differs clearly from the order seen during theta bursts (Fig. 7*A*, lower plot).

To assess the conservation of sequences across the entire dataset, we calculated the correlation between signed imaginary coherence values in wake and sleep periods at each frequency for all cell pairs in both animals (Fig. 8*B*). Because pairwise coherence values are not statistically independent samples, the significance of these correlation coefficients was tested using shuffling (over neurons rather than neuron pairs; see Materials and Methods) to bootstrap the 95% confidence interval (gray lines).

Statistically significant similarity between waking and sleeping sequential relationships was observed across the delta band in both animals. The bottom panels of Figure 8*B* show scatter plots of wake/sleep imaginary coherence between all cell pairs in each animal for an example frequency of 2 Hz.

We also repeated this analysis for the subset of 60 windows in wake and sleep characterized by high LFP theta power (Fig. 8*C*). In this case, significant sequential similarity between wake and sleep was restricted to only the theta frequency. The bottom panels of Figure 8*C* show scatter plots of wake/sleep imaginary coherence between all cell pairs in each animal at 4 Hz. Finally, we asked whether the sequence of firing during theta bursts was related to the delta-band sequential firing during waking. Figure 8*D* shows the relationship between 4 Hz imaginary coherence during theta bursts in sleep and 2 Hz imaginary coherence during the entire waking period. It can be seen that these sequential relationships are unrelated, suggesting that delta-band sequential firing and theta bursts reflect different phenomena.

### Sequential strength is preserved through first and second halves of the night

**Figure 9.**
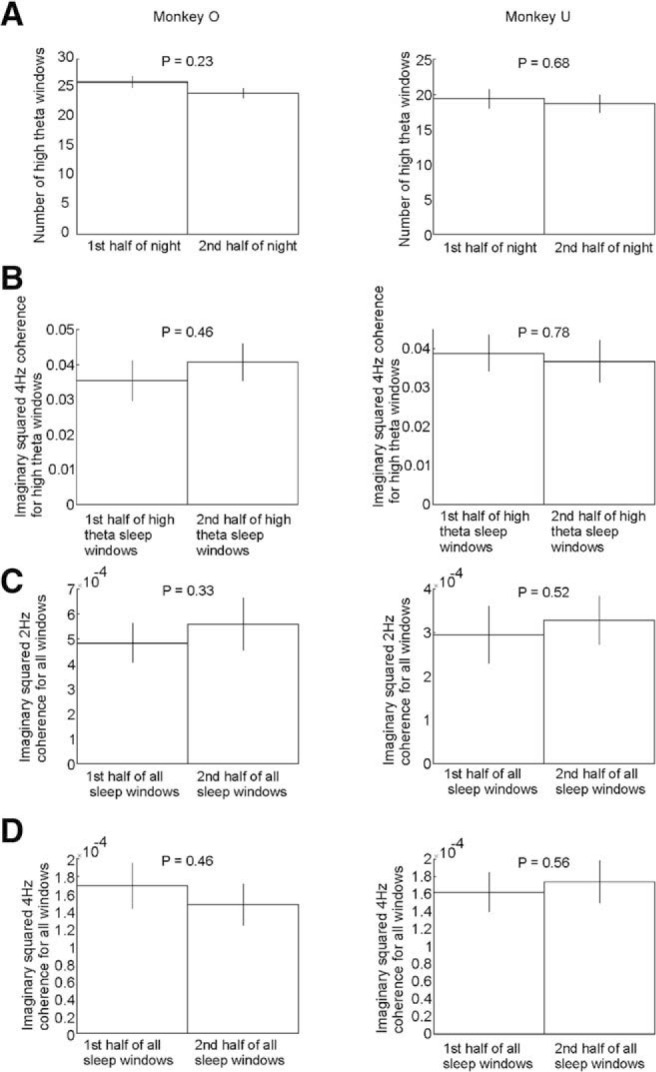
No evidence for change in theta and delta sequence strength during sleep. ***A***, Number of high-power theta windows in first and second half of sleep. ***B***, Mean theta imaginary squared coherence values during theta bursts in the first and second half of sleep. ***C***, Mean delta imaginary squared coherence values in the first and second half of all windows during sleep. ***D***, Mean theta imaginary squared coherence values in the first and second half of all windows during sleep (*P* values from paired *t* test).

We were interested in whether the proportion of theta bursts and/or the strength of sequential activity at either delta or theta frequencies changed systematically through the night. Therefore, we divided the night-time recording into two equal halves and compared the average number of high theta windows that fell within each half. In addition, we used the average imaginary coherence between neuron pairs as a measure of the strength of sequential activity and compared this between first and second halves of the night in the delta and theta bands, as well as for only high-theta windows. We found no significant changes in any of these metrics in either animal (Fig. 9), suggesting that sequences occur equally often and equally strongly throughout the night.

### Inconsistent sequential spike firing during sleep spindles

**Figure 10.**
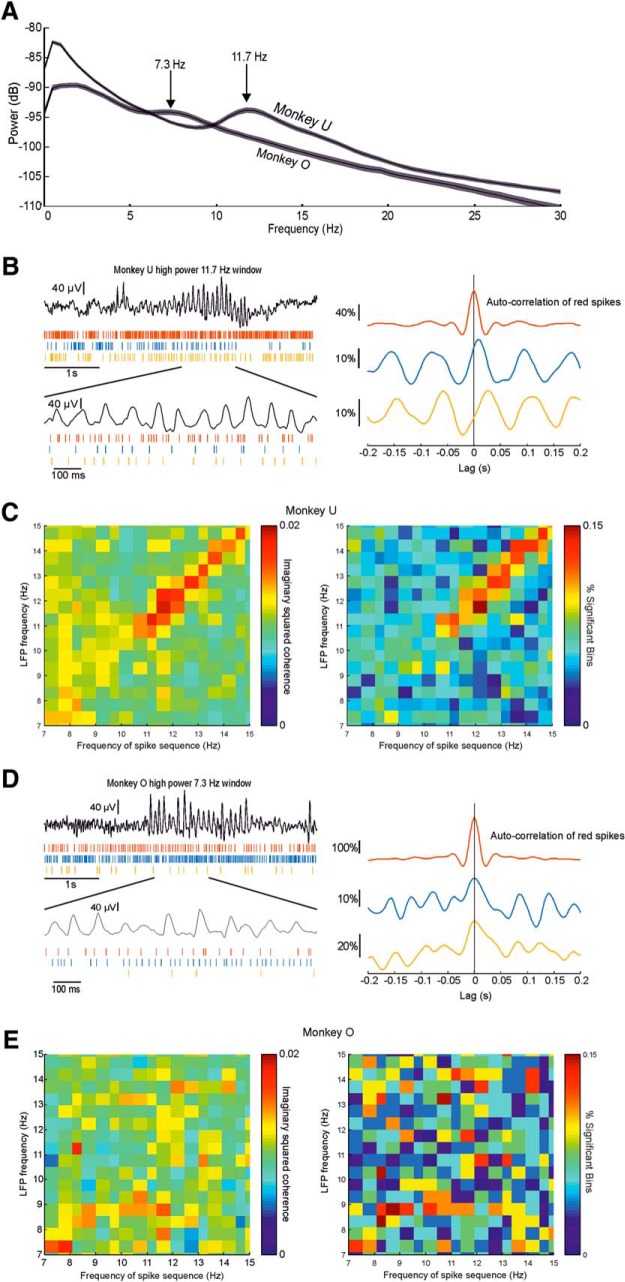
Inconsistent sequential firing during high-power alpha windows. ***A***, Averaged power spectrum of all LFPs in both monkeys during sleep showing peaks at different frequencies in the alpha/sigma-frequency range. ***B***, Left, Example LFP from Monkey U showing putative sleep spindle and concurrent spikes from three neurons. Right, Example CCH showing sequential firing during spindles. ***C***, Left, Mean imaginary squared coherence for all spike pairs during 60 windows with highest relative power at frequencies from 7 to 20 Hz during sleep for Monkey U. Right, Proportion of sessions exhibiting significant imaginary coherence for Monkey U. ***D***, Left, LFP segment from Monkey O showing putative sleep spindle and concurrent spike firing. Right, Example CCH showing only synchronous firing during spindles. ***E***, Left, Mean imaginary squared coherence for all spike pairs during highest 60 windows with highest relative power at frequencies from 7 to 20 Hz during sleep for Monkey O. Right, Proportion of sessions exhibiting significant imaginary coherence for Monkey O.

The LFP during lighter sleep states contained clear spectral peaks at frequencies close to the alpha/sigma band (7–15 Hz; Fig. 3*C,D*) and neural firing was phase-locked to this rhythm (Fig. 5*C*). We were therefore interested in whether there was evidence for sequential firing associated with these putative sleep spindles. The peak frequency was slightly different in each animal, as revealed by the averaged power spectrum of LFPs in Figure 10*A*. Therefore, to isolate epochs most likely to contain sleep spindles, we selected windows with proportionally high power at these specific frequencies (7.3 Hz for Monkey O;11.7 Hz for Monkey U). Figure 10*B* (left) shows an example event recorded from Monkey U, in which a burst of high spindle-frequency LFP is associated with rhythmic firing in three neurons. CCHs for 60 such windows show that these neurons fire in sequence (Fig. 10*B*, right). We applied the same approach as previously to select windows associated with high proportional LFP power at a range of frequencies between 7 and 15 Hz. Across all the data in Monkey U, there was a clear peak in imaginary coherence at ∼12 Hz when windows with high LFP power at this frequency were isolated (Fig. 10*C*). Note, however, that the magnitude of imaginary coherence was substantially lower than at theta frequencies, and statistically significant in only ∼15% of sessions. In Monkey O, we did not observe consistent sequential spiking at any frequency in the spindle range. Figure 10*D* shows an example spindle event with associated neural firing. In this case, the pattern of correlation between neurons was synchronous. Analysis across all sessions revealed no clear or significant imaginary coherence within the spindle band. Therefore, we conclude that sequential spiking during sleep spindles is at best a rare and inconsistent phenomenon that varied across animals.

## Discussion

### Two distinct patterns of sequential firing in motor cortex during sleep

Despite good evidence for a role in sleep rhythms in procedural learning (Nitsche et al., 2010; Walker et al., 2002), there have been few detailed studies on the structure of motor cortical activity during sleep (but see Ramanathan et al., 2015). Studies in nonhuman primates have mainly focused on neuronal correlations during awake motor tasks in which the dominant pattern is synchronous discharge in the beta band (Baker et al., 2001; Jackson et al., 2003) and sequential activity at lower frequencies (Churchland et al., 2012), while observations of precise firing sequences (Prut et al., 1998) have subsequently been questioned (Baker and Lemon, 2000). Hoffman and McNaughton (2002) observed sequential reactivation of neural ensembles in monkeys during rest following a maze task but did not study sleep. Our principal aim was therefore to examine correlations between motor cortical neurons at different frequencies across the sleep cycle, and relate these patterns to those seen during waking behavior.

We quantified sequential activity as consistent, nonzero phase relationships between oscillatory firing patterns (measured by imaginary spike–spike coherence), and therefore cannot discount the further presence of nonoscillatory sequences. Nevertheless, using long datasets recorded in monkeys during natural sleep, we were able to identify two distinct patterns of sequential activity, at delta and theta frequencies, occurring at different phases of the sleep cycle. These may correspond to “slow” and “fast” modes of oscillatory dynamics that have been observed across multiple memory systems (Headley and Paré, 2017).

### Delta sequences

During slow-wave sleep, we observed sequential activation in the delta band as neurons entered cortical up states in a conserved order. The sequential order (established by the sign of imaginary coherence between neuron pairs) was significantly similar to that seen at 2–3 Hz during awake behavior. The peak in imaginary coherence around 2–3 Hz corresponds to the frequency of movement-related cyclical dynamics observed in two-dimensional projections of the high-dimensional trajectories of neural firing (Churchland et al., 2012) and LFPs (Hall et al., 2014a). Note that imaginary coherence and rotational state-space dynamics are essentially the same phenomenon; state-space rotation implies that neurons are consistently active at different phases of a cycle and therefore exhibit imaginary coherence. Conversely, a consistent nonzero phase difference between neurons yields consistently rotating trajectories in state-space projections.

Previously, we have shown that the phase structure of such cyclical LFP dynamics is conserved between movement and sleep (Hall et al., 2014a). Our present results extend this finding by demonstrating that the sequential structure of single neurons is similarly constrained across waking and slow-wave sleep. The ubiquity and consistency of these cycles further strengthens the hypothesis that low-frequency dynamics do not reflect any particular sensorimotor context, but are instead an intrinsic property of motor networks. We suggest that the neural activity associated with specific behavior is constrained by this sequential structure, which demarcates many possible patterns within which specific neural strategies are embedded. It is interesting to note that a similar theory has already been proposed in the sensory domain (Luczak et al., 2009).

### Theta sequences

Our second finding was more unexpected: namely, that motor cortex neurons fire in strikingly regular sequences during brief bursts of theta-band oscillations. These bursts were a rare but robust and statistically significant phenomenon in both animals, clearly evident in the raw recordings.

In rodents, theta oscillations are normally associated with the hippocampus and implicated in spatial navigation and memory (Buzsáki, 2002; Colgin, 2013). Tonic theta oscillations are observed during exploration, and the sequence of place cell firing within the theta cycle is thought to represent the temporal order of experience (Gupta et al., 2012). Theta is rarely investigated in motor areas, although a previous study has described sequential activity at theta frequencies in rodent motor cortex during reaching behavior (Igarashi et al., 2013), possibly reflecting the coupling of hippocampal theta to limb movements (Semba and Komisaruk, 1978). Theta oscillations are also observed in the hippocampus during REM sleep (Colgin, 2013), during which place cells fire in sequences that resemble those seen in awake exploration (Louie and Wilson, 2001). Generally, theta activity is thought to have a role in facilitating communication between areas (Jones and Wilson, 2005) and/or in driving plastic changes to network connectivity via spike timing-dependent plasticity (Sadowski et al., 2016).

In humans, theta oscillations comprise shorter, discrete events, and it is thought that multiple independent mechanisms contribute to the generation of these bursts in the hippocampus and cortex (Cantero et al., 2003). Theta is generally investigated in relation to working-memory (Raghavachari et al., 2001) or spatial-memory tasks (Kahana et al., 1999; Bush et al., 2017) and not generally associated with motor areas. Nevertheless, theta burst stimulation is a reliable way to induce plasticity in the motor cortex (Teo et al., 2011), and some evidence supports a role for REM sleep in the consolidation of procedural memory (Nitsche et al., 2010). Theta oscillations have been observed in prefrontal and anterior cingulate cortices in humans during REM sleep by Vijayan et al. (2017), who suggested roles in consolidation of procedural and emotional memories. Such a hypothesis is supported by our present results, which show that cortical theta in nonhuman primates is associated with sequential neuronal activity similar to that seen in the hippocampus of rodents. The temporal asymmetries ≤∼50 ms that we observed in CCHs (Fig. 7*B*) are within the time window known to drive known spike timing-dependent plasticity mechanisms *in vivo* (Jackson et al., 2006). Interestingly, the sequences of neural firing observed at theta frequencies bore no resemblance to that seen at lower delta frequencies during waking and sleep. These activity patterns would thus seem to reflect a different network state and one that, speculatively, may have appropriate properties to drive neuroplastic changes in motor networks. We were not able to observe a change in sequential strength from the first to the second half of the night. However, further studies involving training on specific behavioral tasks in association with long-term neural recordings may be required to provide evidence for such a role of these theta sequences in neural plasticity motor learning.

### Advantages of long-term recording with a neural data logger

Chronic electrode techniques, such as our moveable microwire arrays, enable the same neurons to be stably monitored long term, allowing comparison between activity patterns during both waking behavior and natural sleep states. However, for nonhuman primates in particular, such experiments require a wireless approach to data recording. One method is to use radio-frequency telemetry, but the wide bandwidth of neural data and large transmission distances within home cages necessitates high power consumption, thus quickly consuming battery life and making long recording impossible (Yin et al., 2014). An alternative solution, as in our present study, is to record data locally on the implant (Mavoori et al., 2005; Zanos et al., 2011). With the growing capacity of commercial nonvolatile memory cards, this approach is increasingly enabling many neurons to be continuously recorded via wide band with only a daily battery recharge. The length of our multineuron datasets proved particularly advantageous in this study. For example, the theta bursts that we found to be associated with sequential firing were observed very rarely and comprised only 0.1–0.2% of the total duration of each recording. Such events might easily be missed in shorter datasets. In future, the number of channels that can be monitored simultaneously may be increased (theoretically by several orders of magnitude) by incorporating spike sorting to compress the data before storage (Luan et al., 2018). We hope these technologies will provide an unprecedented window into how firing is coordinated within distributed neural networks during waking and sleeping.
